# Potential Impact of the Medicare Prescription Payment Plan for Medicare Part D Beneficiaries With a Cancer Diagnosis

**DOI:** 10.1200/JCO-25-01788

**Published:** 2026-01-15

**Authors:** Aryana Sepassi, Scott D. Ramsey, A. Mark Fendrick, Nico Gabriel, Jason A. Zell, Dana B. Mukamel, Sean D. Sullivan

**Affiliations:** ^1^ Department of Pharmacy Practice & Sciences, University of California, San Diego Skaggs School of Pharmacy & Pharmaceutical Sciences, La Jolla, CA; ^2^ Hutchinson Institute for Cancer Outcomes Research, Fred Hutchinson Cancer Center, Seattle, WA; ^3^ Department of Internal Medicine, Division of General Medicine, University of Michigan, School of Medicine, Ann Arbor, MI; ^4^ Department of Medicine, School of Medicine, Chao Family Comprehensive Cancer Center, University of California, Irvine, Irvine, CA; ^5^ Department of Medicine, Division of Internal Medicine, iTEQC Research Program, University of California, Irvine, Irvine, CA; ^6^ The Comparative Health Outcomes, Policy, and Economics Institute, University of Washington, School of Pharmacy, Seattle, WA

## Abstract

**PURPOSE:**

To address high out-of-pocket (OOP) medication costs among Medicare Part D beneficiaries, the 2022 Inflation Reduction Act introduced the Medicare Prescription Payment Plan (M3P), a voluntary program that allows beneficiaries to spread OOP costs over the calendar year. We examined M3P's potential impact among beneficiaries with cancer, who frequently incur substantial early-year Part D medication costs.

**MATERIALS AND METHODS:**

We evaluated a 2022 5% random sample of Medicare beneficiaries with a cancer diagnosis and ≥1 fill for a cancer-indicated Part D medication. We estimated 2025-adjusted annual true OOP spending and median monthly beneficiary OOP payment obligations with and without M3P enrollment. Subgroup analyses were performed by demographics, nonadherence status in 2022, and Part D benefit phase.

**RESULTS:**

Among 168,480 beneficiaries with cancer, most were diagnosed with breast (47.6%), dermatologic (17.6%), or prostate (13.3%) cancers. Overall, 46.7% were projected to reach catastrophic coverage in 2025, with 32.4% doing so in January. Breast (29.7%), prostate (21.20%), and hematologic (20.0%) cancers were most common among those reaching catastrophic coverage. Overall, 43.0% were nonadherent to cancer-indicated Part D medications, with 31.5% reaching catastrophic coverage in January 2025. M3P reduced beneficiary payment obligation variability, especially among those reaching the catastrophic phase in January (IQR, $1,798 US dollars [USD] no M3P *v* $118 USD M3P). Of those reaching catastrophic coverage with a cancer-indicated drug (58.6% overall), 89.2% did so in January.

**CONCLUSION:**

Early-year entry into catastrophic coverage is common among beneficiaries with certain high-cost cancers. M3P may most effectively reduce financial burden when enrollment occurs before January. Targeted outreach from cancer care teams and Part D plans to nonadherent patients and those considering costly therapies could maximize program impact and improve treatment outcomes.

## INTRODUCTION

In 2024, 55% of new cancer diagnoses in the United States occurred in individuals of Medicare-eligible age (≥65 years).^1^ Despite increasing use of cancer screening, improved diagnostics, and more effective therapies, cancer remains a leading cause of death among beneficiaries.^2,3^ With the development of cancer treatments that extend survival and mitigate death, cancer-related expenditures have dramatically increased. By 2030, cancer spending is projected to reach $245.6 billion US dollars (USD) in the United States, including $24.9 billion USD for Medicare Part D medications.^4^

**CONTEXT**

**Key Objective**
How will enrollment in the Medicare Prescription Payment Plan (M3P) affect monthly out-of-pocket (OOP) costs for Medicare beneficiaries with cancer using Part D-covered drugs?
**Knowledge Generated**
Nearly half of the beneficiaries with cancer are expected to reach the OOP cost cap in 2025, with one third doing so in January. Most beneficiaries reaching the OOP cost cap had a breast, prostate, or hematologic cancer diagnosis. Most of the top 20 drugs shifting beneficiaries into the catastrophic phase were associated with a cancer-indicated Part D drug, the most prevalent of which were enzalutamide, abiraterone, ibrutinib, and palbociclib.
**Relevance *(S.B. Wheeler*** *)*
Although M3P does not reduce total annual OOP responsibility for beneficiaries, it does convert often-unaffordable one-time oncology drug payments into predictable installments, smoothing cash flow and mitigating the risk of cost-related nonadherence. Enrollment in M3P, particularly for those beneficiaries expected to reach the OOP cost cap in the first quarter, is important to consider for patients receiving high-cost cancer drugs.*
*Relevance section written by *JCO* Associate Editor Stephanie B. Wheeler, PhD, MPH.

Up to 56% of Medicare beneficiaries with a cancer diagnosis use treatments covered by the Part D benefit, which includes outpatient prescription medications.^5^ Compared with those using cancer-indicated medications under Part B (eg, physician-administered), the out-of-pocket costs are almost three times higher for those using treatments covered under Part D.^5^ Beneficiaries with cancer using Part D cancer-indicated medications in particular often face unpredictably substantial one-time costs that can precipitate financial distress and reduce willingness to initiate or continue treatments.^6-8^ Because of the inverse relationship between cancer-indicated medication costs and adherence, interventions aimed at improving the affordability of Part D medications have the potential to improve adherence and subsequent clinical outcomes among Medicare beneficiaries with cancer.^9-13^

To improve medication affordability and adherence, the Inflation Reduction Act of 2022 included two Part D–related provisions starting in 2025. First, an annual $2,000 USD cap adjusted each year for inflation on true out-of-pocket (TrOOP) spending with zero beneficiary cost-sharing once the limit is reached, and second, the option to voluntarily enroll in the Medicare Prescription Payment Plan (M3P).^14^ Under M3P, Part D plans finance the upfront cost of prescription medications at the pharmacy. Beneficiaries repay plans in zero-interest monthly installments on the basis of remaining months, ongoing costs, and previous balances. Once a beneficiary reaches the catastrophic coverage phase, they no longer pay additional out-of-pocket costs and are responsible for paying any outstanding balance carried over through the end of the year. Although M3P does not reduce total costs, it prevents large one-time payments—up to $2,000 USD—for beneficiaries on limited, fixed incomes.^15^

The Centers for Medicare & Medicaid Services (CMS) estimates that 2.4 million (6.0%) of Part D beneficiaries could benefit from M3P.^16^ Despite the potential financial relief offered by M3P, underenrollment may limit program impact, with only 0.4% of Part D beneficiaries enrolled as of February 2025—the second month of program implementation.^16^ Our primary aim was to estimate the potential beneficiary out-of-pocket payment obligations under the $2,000 USD Part D cap in 2025 using 2022 Medicare claims data, comparing scenarios with and without enrollment in M3P. As a secondary aim, we sought to identify which beneficiaries with cancer would benefit the most from enrollment. We hypothesized that beneficiaries reaching the $2,000 USD cap (catastrophic phase) early in the year would experience the greatest benefit, potentially reducing cost-related nonadherence. As an exploratory analysis, we examined claims that moved beneficiaries into the catastrophic phase under the $2,000 USD Part D cap.

## MATERIALS AND METHODS

### Cohort Identification

We included Part D beneficiaries who were continuously enrolled from January 1, 2022, to December 31, 2022, from a national 5% random sample of Medicare beneficiaries who had a prevalent cancer diagnosis using inpatient, outpatient, and physician claims, and at least one 2022 claim for a Part D cancer-indicated medication after diagnosis (Data Supplement, Table S1, Method S1, online only). We excluded incident cancer cases by identifying those with a claim containing a cancer diagnosis in 2022 and then excluded those without any cancer-related claims 12 months before their first 2022 cancer claim. We excluded incident cases to model enrollment in M3P beginning in January, a decision that we assumed would be plausibly made by beneficiaries already aware of their ongoing need for high-cost cancer therapies. Diagnoses were categorized as breast, dermatologic, GI, hematologic, lung, miscellaneous, prostate, or reproductive cancers (Data Supplement, Table S2). Diagnosis codes for secondary, in situ, or benign cancers were excluded. If a beneficiary had more than one type of cancer diagnosis, then the beneficiary was assigned to a category corresponding to the indication associated with their cancer-indicated Part D medication. We excluded those with any Part C (Medicare Advantage) coverage because of lack of complete medical claims data, and those with mid-year Part D plan switches, which terminate M3P enrollment.^14^ We excluded beneficiaries in plans who were not expected to benefit from M3P, including low-income subsidy (LIS), Medicare-Medicaid dual eligible, and Employer Group Waiver Part D plans.^17^ We included beneficiaries who died in 2022 and calculated costs until death. This study was approved as exempt by the University of California, San Diego Institutional Review Board as only deidentified data were used.

### True Out-of-Pocket Spend Estimation (no M3P scenario)

We used previously published methods to extract and inflate all 2022 Part D claims to 2025 estimates using a ratio derived from the 2021 Medicare Trustee's Report.^17,18^ On the basis of annual percentage increases in Part D per capita costs, we estimated a 1.15 ratio of 2022-to-2025 projected spend.^18^ We estimated each beneficiary's annual TrOOP spend in 2025, defined as the amount that is paid toward eligible Part D prescription medications. Although beneficiary payments (eg, copayments, coinsurance) constitute most TrOOP spend, other spending sources may factor into the total, including plan supplemental benefit payments for enhanced alternative Part D plans (Data Supplement, Method S2), and other TrOOP payments from other third-party payers such as qualified pharmacy assistance programs or charities.^14^ As TrOOP spend accumulates through the calendar year, beneficiaries pass into different coverage phases (Data Supplement, Fig S1). For each beneficiary, we estimated out-of-pocket payment obligations and total annual and monthly TrOOP spend.

### Estimation of Monthly Payment Obligations Under M3P

We used methods published by CMS to simulate monthly beneficiary out-of-pocket payment obligations under M3P (Data Supplement, Method S3).^14^ We assumed all beneficiaries would enroll starting January 2025 and that no beneficiaries would disenroll during the year because of change of plans, missing a late payment, or electing to leave the program.

### Statistical Analysis

We evaluated three main outcomes: (1) monthly beneficiary out-of-pocket payment obligations under the $2,000 USD Part D cap with and without enrollment in M3P, (2) variability in monthly payment obligations across the calendar year in each scenario, and (3) the distribution of nonadherent beneficiaries by their final projected 2025 Part D benefit phases. Beneficiaries were categorized into groups according to the Part D benefit phase each beneficiary ended the calendar year in, as follows: (1) deductible phase (annual TrOOP spend: $0-$591 USD); (2) initial phase (annual TrOOP spend: $591-$1,999 USD); and (3) catastrophic phase (annual TrOOP spend ≥ $2,000 USD). Medication adherence was measured using the proportion of days covered (PDC) and was defined as PDC ≥ 80% (Data Supplement, Method S4). Descriptive statistics for age (in years), sex, racial/ethnic status, region, county-level socioeconomic status, cancer type, and Enhanced Alternative plan enrollment were summarized across all groups using frequencies with proportions. County-level socioeconomic status was derived using Social Vulnerability Index quartiles.^19^ Median, IQR, and maximum beneficiary out-of-pocket payment obligations were summarized for each month under the M3P and no M3P scenarios. Month of advancement to the catastrophic phase was summarized descriptively. Outcomes also were summarized by cancer type. For our exploratory analysis, we summarized the top 20 most frequent medications listed on claims advancing beneficiaries to the catastrophic phase. We also summarized beneficiaries with at least one projected claim in 2025 with an out-of-pocket obligation ≥$600 USD, the threshold CMS used to identify high-cost beneficiaries at the pharmacy for M3P outreach. Sample size estimates were multiplied by 20 to approximate the total Medicare population sample affected from the 5% sample. All statistical analyses were performed using R (R Foundation for Statistical Computing, Vienna, Austria) in R Studio (Posit, Boston, MA).

## RESULTS

A raw total of 8,424 beneficiaries met inclusion criteria, representing 168,480 beneficiaries in the total 2022 Traditional Medicare population (Table 1, Data Supplement, Table S3). Overall, 5.9% (n = 10,000) of beneficiaries died during follow-up. Breast cancer was the most common diagnosis (n = 80,260, 47.6%), followed by dermatologic (n = 29,580, 17.6%) and prostate cancers (n = 22,420, 13.3%). Approximately 23.7% of beneficiaries ended 2025 in the deductible phase (n = 39,940), 29.6% in the initial coverage phase (n = 49,820), and almost half in the catastrophic phase (46.7%, n = 78,720). Overall, 43.0% (n = 72,420) were nonadherent to cancer-indicated medications in 2022. The cancer types with the greatest proportions of nonadherent beneficiaries were dermatologic (78.6%), GI (62.0%), and miscellaneous (54.6%, Data Supplement, Table S4) cancers. Among nonadherent beneficiaries, 22.5% (n = 16,320) were projected to end 2025 in the deductible phase, 29.6% (n = 21,440) in the initial coverage phase, and 47.9% (n = 34,660) in the catastrophic phase. Nonadherent beneficiaries were expected to reach the catastrophic phase in a median 3.0 months (IQR, 0.0-5.0 months).

**TABLE 1.** Demographic Characteristics

Characteristic	Total^a^ (N = 168,480), No. (%)	Deductible Phase^b^ (n = 39,940), No. (%)	Initial Phase (n = 49,820), No. (%)	Catastrophic Phase (n = 78,720), No. (%)
Age, years				
65-74	77,360 (45.9)	22,160 (55.5)	22,380 (44.9)	32,820 (41.7)
75-84	68,600 (40.7)	13,860 (34.7)	20,640 (41.4)	34,100 (43.3)
85+	22,520 (13.4)	3,920 (9.8)	6,800 (13.6)	11,800 (15.0)
Sex				
Female	109,800 (65.2)	32,280 (80.8)	36,740 (73.7)	40,780 (51.8)
Male	58,680 (34.8)	7,660 (19.2)	13,080 (26.3)	37,940 (48.2)
Race/ethnicity				
NH, White	152,600 (90.6)	36,400 (91.1)	46,340 (91.0)	70,860 (90.0)
NH, Black	4,940 (2.9)	1,120 (2.8)	1,220 (2.4)	2,600 (3.3)
Hispanic	3,020 (1.8)	760 (1.9)	1,040 (2.1)	1,220 (1.5)
Other	7,920 (4.7)	1,660 (4.2)	2,220 (4.5)	4,040 (5.1)
Region				
South	66,220 (39.3)	15,320 (38.4)	21,080 (42.3)	29,820 (37.9)
Midwest	37,580 (22.3)	9,180 (23.0)	10,600 (21.3)	17,800 (22.6)
West	32,880 (19.5)	8,520 (21.3)	9,480 (19.0)	14,880 (18.9)
Northeast	31,800 (18.9)	6,920 (17.3)	8,660 (17.4)	16,220 (20.6)
Area-level socioeconomic status (quartile)				
First (highest)	41,460 (24.6)	9,740 (24.4)	12,260 (24.6)	19,460 (24.7)
Second	41,860 (24.8)	10,400 (26.0)	12,400 (24.9)	19,060 (24.2)
Third	41,060 (24.4)	9,880 (24.7)	11,960 (24.0)	19,220 (24.4)
Fourth (lowest)	44,100 (26.2)	9,920 (24.8)	13,200 (26.5)	20,980 (26.7)
Cancer type				
Breast	80,260 (47.6)	27,700 (69.4)	29,160 (58.5)	23,400 (29.7)
Dermatologic	29,580 (17.6)	6,580 (16.5)	10,800 (21.7)	12,200 (15.5)
GI	3,160 (1.9)	560 (1.4)	660 (1.3)	1,940 (2.5)
Hematologic	18,980 (11.3)	860 (2.2)	2,380 (4.8)	15,740 (20.0)
Lung	4,180 (2.5)	580 (1.5)	760 (1.5)	2,840 (3.6)
Miscellaneous	6,700 (4.0)	740 (1.9)	1,440 (2.9)	4,520 (5.7)
Prostate	22,420 (13.3)	2,100 (5.3)	3,660 (7.3)	16,660 (21.2)
Reproductive	3,200 (1.9)	820 (2.1)	960 (1.9)	1,420 (1.8)
Plan type				
Enhanced alternative	138,020 (81.9)	32,100 (80.4)	41,580 (83.5)	64,340 (81.7)
Died	10,000 (5.9)	1,540 (3.9)	1,820 (3.7)	6,640 (8.4)
Nonadherent^c^	72,420 (43.0)	16,320 (40.9)	21,440 (43.0)	34,660 (44.0)

Abbreviations: NH, non-Hispanic; USD, US dollars.

^a^ Raw sample sizes were multiplied by 20 to approximate the total Traditional Medicare population sample on the basis of the 5% random sample used for analysis. A version of Table 1 containing raw sample sizes can be found in the Data Supplement (Supplementary Materials).

^b^ Groups were defined according to total estimated true out-of-pocket spend in 2025 with the Inflation Reduction Act's Part D cap in place. Deductible: $0-590 USD; initial phase: $591-1,999 USD; catastrophic: $2,000 USD.

^c^ Defined as percent of days covered <80% to cancer-indicated Part D medications in calendar year 2022.

**FIG 1.**
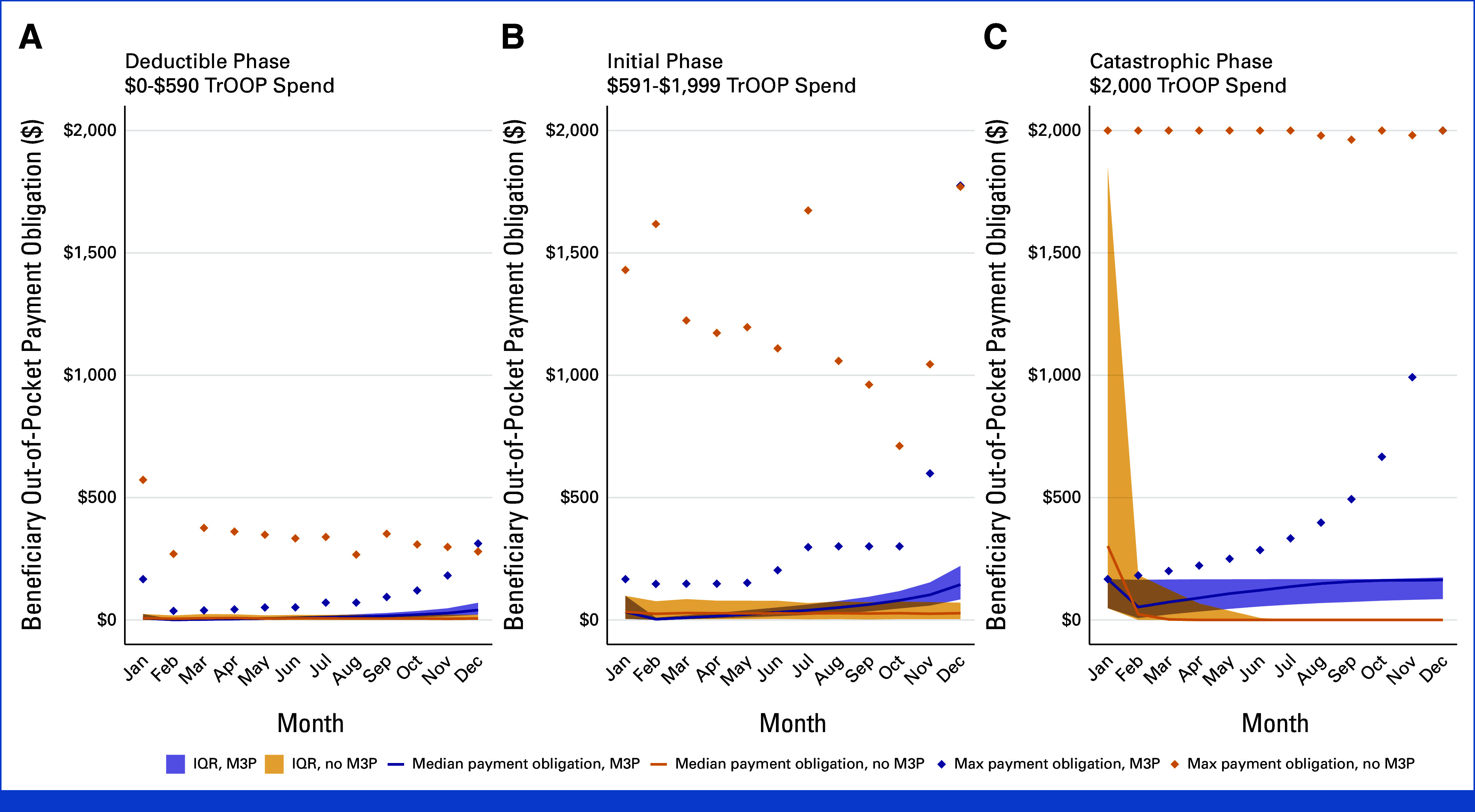
Monthly beneficiary out-of-pocket payment obligations with and without enrollment in the Medicare Prescription Payment Plan by projected Part D phase, 2025. Median monthly beneficiary payment obligations under the M3P and without the M3P are denoted by solid and dotted black lines, respectively. Monthly IQRs (variation) of median beneficiary out-of-pocket payment obligations for the M3P and no M3P scenarios are denoted by blue and orange shading, respectively. The maximum beneficiary payment obligations observed for each month under the M3P and no M3P scenarios are denoted using blue and orange diamonds, respectively. Beneficiaries were assigned to each of the study groups according to estimated total true out-of-pocket spend in 2025. M3P, Medicare Prescription Payment Plan.

Estimated median monthly beneficiary out-of-pocket payment obligations and their variability (IQR) with and without M3P enrollment are shown in Figure 1. Maximum monthly payment obligations were lower under the M3P scenario for all groups and months, except in December (Data Supplement, Tables S5-S7). The greatest reduction in monthly payment obligation variability occurred in January among beneficiaries who reached the catastrophic phase (no M3P IQR, $1,798 USD; M3P IQR, $118 USD). For this group, median monthly payment obligations under M3P were higher than those under No M3P beginning in February; however, payment obligations were more evenly distributed during the year. Those with a hematologic cancer, on average, demonstrated the greatest financial frontloading without M3P, where 55.4% of total annual out-of-pocket payment obligations were estimated to be paid in January.

**FIG 2.**
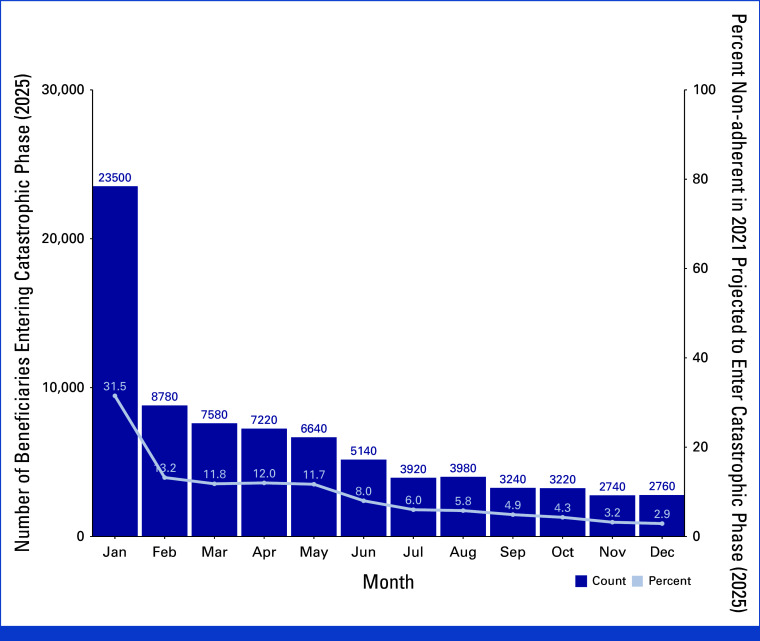
Beneficiaries projected to reach the catastrophic phase in 2025 and percent nonadherent to cancer-indicated Part D medications in 2022 by month. Dark blue bars represent the total number of beneficiaries projected to reach the catastrophic phase in 2025 by month (January-December). The percentage of beneficiaries who were nonadherent to cancer-indicated Part D medications in 2022 projected to reach the catastrophic phase in 2025 is denoted by the overlaid light blue line. Sample sizes were multiplied by 20 to estimate the total Traditional Medicare population size affected from the 5% random sample used for analysis. M3P, Medicare Prescription Payment Plan; TrOOP, true out-of-pocket cost.

Of those projected to reach the catastrophic phase at any time during 2025, the greatest proportion (n = 23,500, 32.4%) reached the $2,000 USD TrOOP threshold in January (Fig 2). Approximately one third of the 74,420 beneficiaries who were nonadherent to a cancer-indicated Part D medication were projected to reach the catastrophic phase in 2025 in January (n = 22,812, 31.5%). Breast cancer diagnoses were the most common among those expected to reach the catastrophic phase in 2025 (n = 23,400, 29.7%), followed by prostate (n = 16,660, 21.2%) and hematologic cancers (n = 15,740, 20.0%), representing approximately 1.1% of all Traditional Medicare beneficiaries with cancer.^20,21^

**FIG 3.**
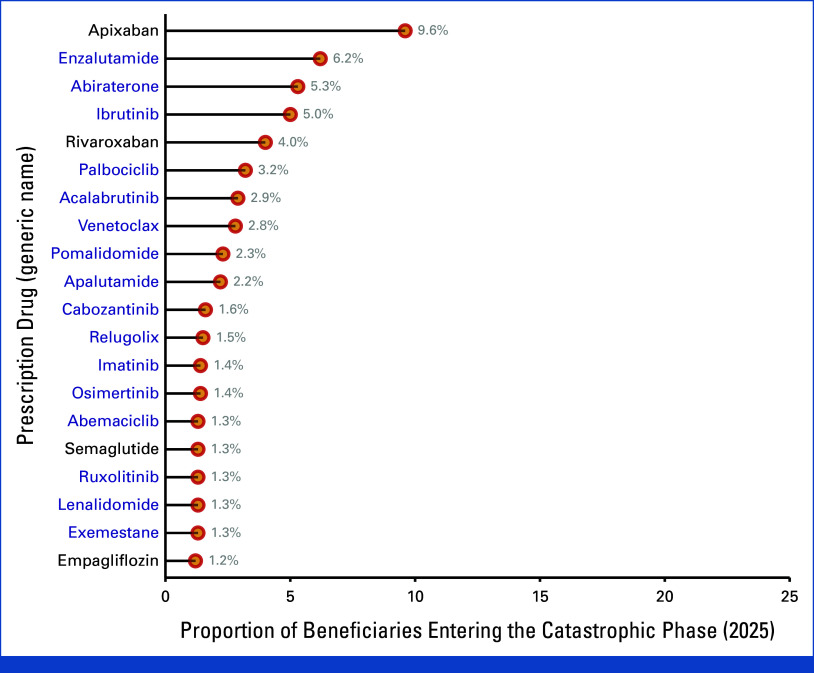
Top 20 most frequently presenting Part D medications on claims advancing beneficiaries with cancer to the catastrophic phase, 2025. The proportion of beneficiaries reaching the catastrophic phase with each listed drug is denoted in light gray. Cancer-indicated Part D medications are denoted in blue text; non–cancer-indicated Part D drugs are denoted in black along the *y*-axis.

Among beneficiaries projected to reach the catastrophic phase in 2025, over half (n = 40,240, 58.6%) had a single cancer-indicated medication listed on the Part D claim that triggered entry into the catastrophic phase. Of these beneficiaries, 89.2% of those who reached catastrophic coverage in January did so with a cancer-indicated Part D medication (n = 20,960). Of the 20 most frequently observed medications linked to claims that advanced beneficiaries into the catastrophic phase, 16 were cancer-indicated (Fig 3). Enzalutamide was the most common cancer-indicated Part D medication in this group, representing 4,880 beneficiaries (6.2%) in the total Medicare population of all beneficiaries projected to reach catastrophic coverage, followed by abiraterone (n = 4,172, 5.3%) and ibrutinib (n = 3,936, 5.0%). Nearly all beneficiaries with a projected out-of-pocket claim obligation ≥$600 USD were in the catastrophic phase (n = 47,560, 95.2%, Data Supplement, Table S8).

## DISCUSSION

Our findings suggest that Medicare beneficiaries with cancer who reach the catastrophic phase—especially the one third who do so in January—may gain the most from M3P enrollment. With $0 USD beneficiary cost-sharing upon reaching the catastrophic phase, M3P enrollment early in the year would allow beneficiaries to pay their out-of-pocket payment obligations in fixed and predictable monthly installments. Although M3P does not reduce total annual out-of-pocket responsibility for beneficiaries, it alters the timing and distribution of these costs. Historically, large and unpredictable lump-sum costs have created financial distress for Part D beneficiaries with cancer and may have served as a treatment barrier.^6-8^ In response to this financial burden, M3P converts these often-unaffordable one-time payments into predictable monthly installments, smoothing cash flow and potentially mitigating the risk of cost-related nonadherence. This financial predictability, especially for early-year catastrophic entrants, mitigates cost shocks and may preserve critical cancer care access. Elimination of beneficiary cost-sharing in the catastrophic phase starting in 2024 has been associated with a 53% increase in volumes of oncology prescriptions, and beneficiaries are expected to experience a reduction in overall TrOOP spend with the $2,000 USD cap starting in 2025.^22,23^ Enrollment in M3P offers an additional layer of financial protection to these benefits that may be particularly favorable for those reaching the $2,000 USD cap early in the year.

Our findings support CMS's mandate that Part D plans and pharmacies prioritize outreach for high-cost beneficiaries when promoting M3P enrollment. Consistent with previous findings, we found that most beneficiaries reaching $2,000 USD in out-of-pocket spending did so early in the year.^24,25^ Notably, most of these individuals had a breast, prostate, or hematologic cancer diagnosis. Although these beneficiaries are most likely to currently benefit from M3P enrollment, advances in cancer therapeutics may shift which subgroups are most likely to benefit from M3P in the future. Our findings should be interpreted as reflecting the therapeutic landscape of 2022, with recognition that the profile of high-cost beneficiaries may evolve over time.

Nearly one third of those nonadherent to Part D cancer-indicated medications in 2022 also reached the $2,000 USD cap early in the year. Higher expected out-of-pocket costs reduce initiation and adherence to high-cost medications among Part D beneficiaries.^9-13^ Estimates suggest 16.5% of beneficiaries with cancer report cost-related nonadherence, with greater odds among Traditional Medicare and non-LIS Part D beneficiaries, and 30% of non-LIS beneficiaries do not initiate high-cost Part D cancer medications, presumably because of cost.^26,27^ If beneficiaries are aware of the $2,000 USD cap and M3P in advance of a new calendar year, adherence may improve because they will face no further cost-sharing after the threshold. For the one third who reach the cap early in the year, the option to spread costs through M3P maximizes benefit by preventing large upfront financial shocks. Preemptive enrollment and education for these individuals could improve not only adherence but also downstream clinical benefits. Tailored implementation strategies will be essential to ensure these benefits are equitably realized, particularly among vulnerable populations at greater risk of poor cancer outcomes. However, while we expect early attainment of the catastrophic phase will maximize the benefit of M3P for some, most nonadherent beneficiaries who reached the catastrophic phase did not do so in January—highlighting the need for complementary strategies beyond M3P to mitigate financial distress over the year. The benefits M3P offers for nonadherent individuals may differ for those who were adherent in 2022 and reached the $2,000 USD cap in January. Although improvements to adherence may not be as likely for these individuals, M3P may still aid in preserving disposable income for other needs (eg, food insecurity, housing insecurity).

Overall, utilization of novel targeted outreach mechanisms is critical among beneficiaries with cancer likely to reach the catastrophic phase early in the year, as low enrollment rates in M3P highlight a gap in awareness and uptake.^16^ For example, plans may adopt automatic enrollment in M3P at the beginning of the calendar year for early-year high-spend beneficiaries of the year before, with an opt-out option. Using an opt-out method for these beneficiaries before the year starts would guide individuals toward financial protection while still preserving freedom of choice. These types of behavioral nudges have demonstrated positive outcomes for various cancer-related interventions.^28-30^ Proactive educational efforts from providers caring for the high-cost beneficiaries with cancer characterized in our analysis may also improve M3P uptake when considering the risk benefit of treatment options. Informing providers of ideal patient candidates for M3P may be delivered using existing mechanisms (eg, provider-based electronic health record alerts, preemptive plan-provider coordination during the prescribing process). Other members of the cancer care team, such as social workers, pharmacists, and financial navigators, are also well suited to inform beneficiaries about M3P, as they are knowledgeable about effective strategies to minimize out-of-pocket costs and have been shown to reduce the financial burden of care for people with cancer.^31,32^ Approximately half of the people with cancer receiving copayment financial assistance are Traditional Medicare beneficiaries, of whom the majority report learning about copayment assistance from either clinicians or financial navigators.^33^

The current lower-than-expected enrollment in M3P suggests that some beneficiaries with cancer identified in our analysis who would benefit the most are not currently enrolled.^16^ Although additional targeted outreach mechanisms inclusive of the cancer care team could improve enrollment rates, current underenrollment may also reflect current plan-level disincentives. Administration of the program requires additional costs, and plans bear the financial risk if beneficiaries default on installment payments. This risk is compounded by the potential for adverse selection, as those enrolling and benefitting from M3P are more likely to be high-cost users, including beneficiaries with cancer. Without mechanisms to offset uncollected payments, plans may be discouraged from promoting enrollment. To address these concerns, CMS could consider aligning M3P outreach, enrollment, and retention with existing quality performance measures (eg, Star Ratings) or by providing reinsurance for defaulted payments.

Our analysis was subject to limitations. We assumed no changes from 2022 to 2025 in Part D enrollment trends, cancer incidence, beneficiary cost-sharing, drug utilization, and formulary management, nor did we account for other direct or indirect changes to Part D from the IRA (eg, $35 USD flat copayment for insulin, drug price negotiation, inflation rebates, increased premiums/coinsurance, etc).^14^ Exclusion of Part C beneficiaries may limit generalizability of findings. We were unable to determine cancer stage or prognosis because of the nature of claims data, which may have affected the sensitivity of cancer case identification. Exclusion of incident cases and potential adherence misclassification bias for medications not prescribed daily (eg, 3-weeks-on/1-week-off regimens) could have led to underestimation of results. Lung cancer cases were also likely underrepresented in our cohort as only 10% of beneficiaries with a lung cancer diagnosis use Part D-covered treatment.^34^

M3P is a voluntary program that was implemented to provide financial relief for Medicare Part D beneficiaries who incur over $2,000 USD in annual TrOOP spend, with the goal of improving cost-related nonadherence. Given current suboptimal enrollment, targeted outreach should be integrated early into the cancer care continuum by engaging health care providers who can identify beneficiaries who may benefit from enrollment. On the basis of our analysis, additional resources should be devoted to those beneficiaries with hematologic, breast, and reproductive cancer diagnoses, with fewer additional resources expended on those less likely to meaningfully benefit from M3P. The use of a more targeted approach by Part D plans and integration with health care providers would better fulfill the aims of the M3P program to support equitable, continuous access to life-sustaining therapies and fulfill the policy goals of the Inflation Reduction Act.
