# Supplementary material for: Potential Impact of the Medicare Prescription Payment Plan for Medicare Part D Beneficiaries With a Cancer Diagnosis
Source: J Clin Oncol. 2026 Jan 15;44(7):598–606. doi: 10.1200/JCO-25-01788 (PMC12931864; doi:10.1200/JCO-25-01788)
Supplement: Supplementary file 1 [file jco-44-598-s001.pdf]

Supplementary Materials – Table of Contents

Table S1. List of Cancer-Indicated Part D Prescription Medications 2
Method S1. Identification of Cancer-Indicated Part D-Covered Prescription Drugs 6
Table S2. List of ICD-10-CM Cancer Diagnosis Codes and Groupings 7
Method S2. Rationale and Identification of Enhanced Alternative Part D Plans 8
Figure S1. Medicare Part D Benefit Coverage Phases, 2025 9
Method S3. Calculation of Monthly Beneficiary Out-of-Pocket Payment Obligations under the Medicare Prescription Payment Plan (M3P) 10
Method S4. Analysis of Proportion of Days Covered (PDC) as a Measure of Adherence 11
Table S3. Demographic Characteristics, Raw 12
Table S4. Beneficiary Out-of-Pocket Obligations and Adherence by Cancer Type 13
Table S5. Median and Interquartile Range of Monthly Beneficiary Charges, Medicare Prescription Payment Plan (M3P) vs No Medicare Prescription Payment Plan (No M3P), Deductible Phase, 2025 14
Table S6. Median and Interquartile Range of Monthly Beneficiary Charges, Medicare Prescription Payment Plan (M3P) vs No Medical Prescription Payment Plan (No M3P), Initial Phase, 2025 15
Table S7. Median and Interquartile Range of Monthly Beneficiary Charges, Medicare Prescription Payment Plan (M3P) vs No Medical Prescription Payment Plan (No M3P), Catastrophic Phase, 2025 16
Table S8. Demographic Characteristics Among Beneficiaries with 2025 Part D Claims with Out-of-Pocket Obligations of $600 or Greater 17

Table S1. List of Cancer-Indicated Part D Prescription Medications

Brand Name	Generic Name	Brand Name	Generic Name
Abiraterone*	Abiraterone*	Empliciti	Elotuzumab
Abraxane	Paclitaxel	Enhertu	fam-Trastuzumab deruxtecan-nxki
Adectris	Brentuximab vedotin	Erbitux	Cetuximab
Afinitor*	Everolimus*	Erivedge*	Vismodegib*
Alecensa*	Alectinib*	Erleada*	Apalutamide*
Alunbrig	Brigantinib	Erlotinib*	Erlotinib*
Aromasin*	Exemestane*	Etoposide	Etoposide
Avastin	Bevacizumab	Everolimus*	Everolimus*
Ayvakit	Avapritinib	Exemestane*	Exemestane*
Azacitidine	Azacitidine	Exkivity*	Mobocertinib*
Balversa*	Erdafitinib*	Fareston	Toremifene
Bavencio	Avelumab	Farydak	Panobinostat
Bendeka*	Bendamustine*	Faslodex*	Fulvestrant*
Bexarotene*	Bexarotene*	Femara	Letrozole
Blincyto	Blinatumomab	Firmagon	Degarelix
Bortezomib*	Bortezomib*	Fluorouracil*	Fluorouracil*
Bosulif*	Bosutinib*	Flutamide	Flutamide
Braftovi*	Encorafenib*	Fotivda*	Tivozanib*
Brukinsa	Zanbrutinib	Fulvestrant*	Fulvestrant*
Cabometyx*	Cabozantinib*	Gavreto*	Pralsetinib*
Calquence*	Acalabrutinib*	Gazyva*	Obinutuzumab*
Capecitabine	Capecitabine	Gemcitabine	Gemcitabine
Caprelsa	Vandetanib	Gilotrif*	Afatinib*
Carboplatin*	Carboplatin*	Gleevec*	Imatinib*
Cisplatin	Cisplatin	Gleostine	Lomustine
Cometriq	Cabozantinib	Halaven	Eribulin
Copiktra*	Duvelisib*	Herceptin	Trastuzumab
Cotellic*	Cobimetinib*	Herzuma	Trastuzumab-pkrb
Cyclophosphamide*	Cyclophosphamide*	Hydea	Hydroxyurea
Cyramza	Ramucirumab	Hydroxyurea*	Hydroxyurea*
Cytarabine*	Cytarabine*	Ibrance*	Palbociclib*
Dacarbazine	Dacarbazine	Iclusig*	Ponatinib*
Darzalex	Daratumumab	Idhifa*	Enasidenib*
Darzalex Faspro*	Daratumumab-hyalurnoidase*	Imatinib*	Imatinib*
Daurismo*	Glasdegib*	Imbruvica*	Ibrutinib*
Decitabine	Decitabine	Imfinzi	Duravulumab
Docetaxel	Docetaxel	Infugem	Gemcitabine
Doxil*	Doxorubicin*	Inlyta	Axitinib
Doxorubicin	Doxorubicin	Inqovi*	Decitabine / Cedazuridine*
Droxia*	Hydroxyurea*	Iressa	Gefitinib
Efudex*	Fluoruracil*	Irinotecan	Irinotecan
Eligard*	Leuprolide*	Istodax	Romidepsin
Emcyt*	Estramustine*	Ixempra	Ixabepilone

Table S1 (Cont'd):

Brand Name	Generic Name	Brand Name	Generic Name
Jakafi*	Ruxolitinib*	Opdualag*	Nivolumab / Relatlimab*
Jevtana	Cabazitaxel	Opzelura*	Ruxolitinib*
Kadcyla	ado-Trastuzumab	Orgovyx*	Relugolix*
Kanjinti	Tastuzumab-anns	Oxaliplatin	Oxaliplatin
Keytruda*	Pembrolizumab*	Paclitaxel	Paclitaxel
Kisqali*	Ribociclib*	Padcev	Enfortumab vedotin-ejfv
Kyprolis	Carfilzomib	Pemazyre*	Pemigatinib*
Lapatinib	Lapatinib	Perjeta	Pertuzumab
Lemtrada	Alemtuzumab	Phesgo	Pertuzumab / Trastuzumab-hydraluronidase-zzxf
Lenalidomide*	Lenalidomide*	Piqray*	Alpelisib*
Lenvima	Lenvantinib	Polteligeo	Mogamulizumab
Letrozole*	Letrozole*	Pomalyst*	Pomalidomide*
		Prolia*	Denosumab*
Leukeran*	Chlorambucil*	Purixan	Mercaptopurine
Libtayo	Cemiplimab	Qinlock	Ripretanib
Lonsurf*	Trifluridine / Tipracil*	Retevemo*	Selpercatinib*
Lorbrena*	Lorlatinib*	Revlimid*	Lenalidomide*
Lumakras*	Sotorasib*	Rituxan	Rituximab
Lupron*	Leuprolide*	Rozlytrek*	Entrectinib*
Lynparza*	Olaparib*	Rubraca*	Rucaparib*
Lysodren*	Mitotane*	Ruxience	Rituximab-pvvr
Matulane	Procarbazine	Rybrevant	Amivantamab-vmjw
Megestrol*	Megestrol*	Sarclisa	Isatuximab-irfc
Mekinist*	Trametinib*	Scemblix	Asciminib
Mektovi*	Binimetinib*	Soltamox	Tamoxifen
Melphalan	Melphalan	Sprycel*	Dasatinib*
Mercaptopurine*	Mercaptopurine*	Stivarga*	Regorafenib*
Methotrexate*	Methotrexate*	Sorafenib*	Sorafenib*
Mitomycin*	Mitomycin*	Sunitinib*	Sunitinib*
Monjuvi	Tafasitamab-cxix	Sutent*	Sunitinib*
Mvasi	Bevacizumab-awwb	Synribo	Omacetaxine
Myleran	Busulfan	Tabrecta*	Capmatinib*
Nerlynx*	Neratinib*	Tafinlar*	Dabrafenib*
Nexavar*	Sorafenib*	Tagrisso*	Osimertinib*
Nilandron*	Nilutamide*	Talzenna	Talazoparib
Nilutamide*	Nilutamide*	Tamoxifen*	Tamoxifen*
Ninlaro*	Ixazomib*	Tarceva	Erlotinib
Nubeqa*	Darolutamide*	Targretin	Bexarotene
Odomzo	Sonidegib	Tasigna*	Nilotinib*
Ofev*	Nintedanib*	Tecentriq	Atezolizumab
Ogiviri	Trastuzumab-dkst	Tepmetko	Tepotinib
Onivyde	Irinotecan liposome	Thalomid*	Thalidomide*
Onureg*	Azacitidine*	Tibsovo*	Ivosidenib*
Opdivo*	Nivolumab*	Toremifene*	Toremifene*

Table S1 (Cont'd):

Brand Name	Generic Name
Trazimera	Trastuzumab-qyyp
Treanda*	Bendamustine*
Trelstar*	Triptorelin*
Trexall	Methotrexate
Trodelvy	Sacituzumab govitecan-hziy
Truseltiq	Infigratinib
Truxima*	Rituximab-abbs*
Tukysa*	Tucatinib*
Turalio	Pexidartinib
Tykerb	Lapatinib
Ukoniq*	Umbralisib*
Valchlor*	Mechlorethamine*
Valrubicin*	Valrubicin*
Vectibix	Panitumumab
Velcade*	Bortezomib*
Venclexta*	Venetoclax*
Verzenio*	Abemaciclib*
Vinblastine	Vinblastine
Vinorelbine	Vinorelbine
Vitrakvi	Larotrectinib
Vizimpro	Dacomatinib
Votrient*	Pazopanib*
Welireg*	Belzutifan*
Xalkori*	Crizotinib*
Xeloda	Capecitabine
Xgeva*	Denosumab*
Xospata*	Gilteritinib*
Xpovio*	Selinexor*
Xtandi*	Enzalutamide*
Yervoy	Ipilimumab
Yondelis	Trabectidin
Yonsa*	Abiraterone*
Zejula*	Niraparib*
Zelboraf*	Vemurafenib*
Zepzelca	Lurbinectedin
Zirabev*	Bevacizumab-bvzr*
Zoladex	Goserelin
Zolinza	Vorinostat
Zydelig	Idelalisib
Zykadia	Ceritinib
Zytiga*	Abiraterone*

Drugs that were identified in the 5% random sample Part D event (PDE) data are denoted with an asterisk (*). Drugs available as generics are indicated using the same name under both the "Brand Name" and "Generic Name" columns. These drugs may have also had brand-name versions identified. Drugs that were listed and identified in the Part D claims dataset that may also have coverage under the Part B benefit were included for

analysis. For the purposes of adherence calculations, these drugs were confirmed to contain the correct days' supply/cycle length for each claim. Any cancer-indicated drugs that were filled under the Part B benefit were excluded from analysis.

Method S1. Identification of Cancer-Indicated Part D-Covered Prescription Drugs

The list of cancer-indicated Part D-covered prescription drugs that was used to identify and flag claims in the 2022 5% random sample Part D event (PDE) claims (Table S1) was empirically derived using the publicly available Medicare Part D drug spending dashboard (https://data.cms.gov/tools/medicare-part-d-drug-spending-dashboard).[1] The Centers for Medicare and Medicaid Services (CMS) annually collects all Part D event records (claims) and aggregates spending by unique drug, in addition to other metrics, for presentation on the dashboard. The data presented on the dashboard are derived from all Part D organization and plan types, excluding over-the-counter medications and drugs with 11 or fewer claims. Author AS manually reviewed the entire list of drugs on the 2022 dashboard and extracted all drug names that contained a cancer indication. The extracted list of drugs was used to filter the 2022 5% random sample Part D event (PDE) claims data using generic drug name. Not all drugs searched were identified in the dataset.

References:

1. Centers for Medicare and Medicaid Services. Medicare Part D Spending by Drug Methodology. Published 2025. Accessed October 3, 2025. https://data.cms.gov/sites/default/files/2025-05/DSD_PTD_RY25_20250425_Methodology_WDDSE_508.pdf

Table S2. List of ICD-10-CM Cancer Diagnosis Codes and Groupings

Cancer Type	ICD-10-CM Codes
Breast	C50
Dermatologic	C43 – C44
Gastrointestinal	C15 – C26
Hematologic	C81 – C96
Lung	C30 – C39
Miscellaneous[1]	C00 – C14[2], C40-C41, C45-C49, C64-C68[3], C69[4], C70-C72-C75, C7A, C76-C80
Prostate	C61
Reproductive	C51 – C63[5]

Medicare beneficiaries were included in any of the listed cancer categories if they had any of the above listed ICD-10-CM diagnosis codes listed on any one inpatient or two outpatient or physician claims (at most separated by 30 days) for 2022. Beneficiaries with no cancer diagnoses flagged within the year before the first noted claim with a cancer diagnosis code in 2022 were defined as "incident" cases and were excluded from analysis.

[1]Includes malignant neoplasms of bone and articular cartilage, mesothelial and soft tissue, brain, other parts of the central nervous system, neuroendocrine tumors, endocrine malignant neoplasms, malignant neoplasms of the head and neck, and other ill-defined or unspecified sites.
[2]Malignant neoplasms of the lip, oral cavity, and pharynx
[3]Malignant neoplasms of the urinary tract (includes renal cancers)
[4]Malignant neoplasms of the eye
[5]Includes malignant neoplasms of the male and female genital organs (excluding prostate cancer)

Method S2. Rationale and Identification of Enhanced Alternative Part D Plans

Enhanced Alternative (EA) Part D plans offer benefits such as $0 deductibles or coverage of medications not covered by Standard Benefit plans, leading to lower out-of-pocket costs for beneficiaries. Lower out-of-pocket costs for beneficiaries in EA plans may result in delays toward reaching the $2,000 TrOOP cap under the IRA. To avoid disincentivizing enrollment in EA plans as a result, the IRA implemented “greater of” logic in the definition of TrOOP.[1] Beneficiary payments for each Part D claim under an EA plan that are included in TrOOP spend calculations are the greater of either 1) what the beneficiary actually paid under the EA plan or 2) what the beneficiary would have paid had they had a Standard Benefit plan.[1] Because of the “greater of” logic, and because supplemental payments from enhanced Part D plans contribute to TrOOP spend, beneficiaries in EA plans may reach the $2,000 TrOOP cap without spending $2,000 themselves. For each EA beneficiary’s claim, we compared the actual observed beneficiary co-payment under the EA plan to the counterfactual Standard Benefit scenario and applied the greater of the two to the final TrOOP calculation for each claim.

References:

1. Rep. Yarmuth JA [D K 3. Text - H.R.5376 - 117th Congress (2021-2022): Inflation Reduction Act of 2022. Published 2022. Accessed April 17, 2025. https://www.congress.gov/bill/117th-congress/house-bill/5376/text

Figure S1. Medicare Part D Benefit Coverage Phases, 2025

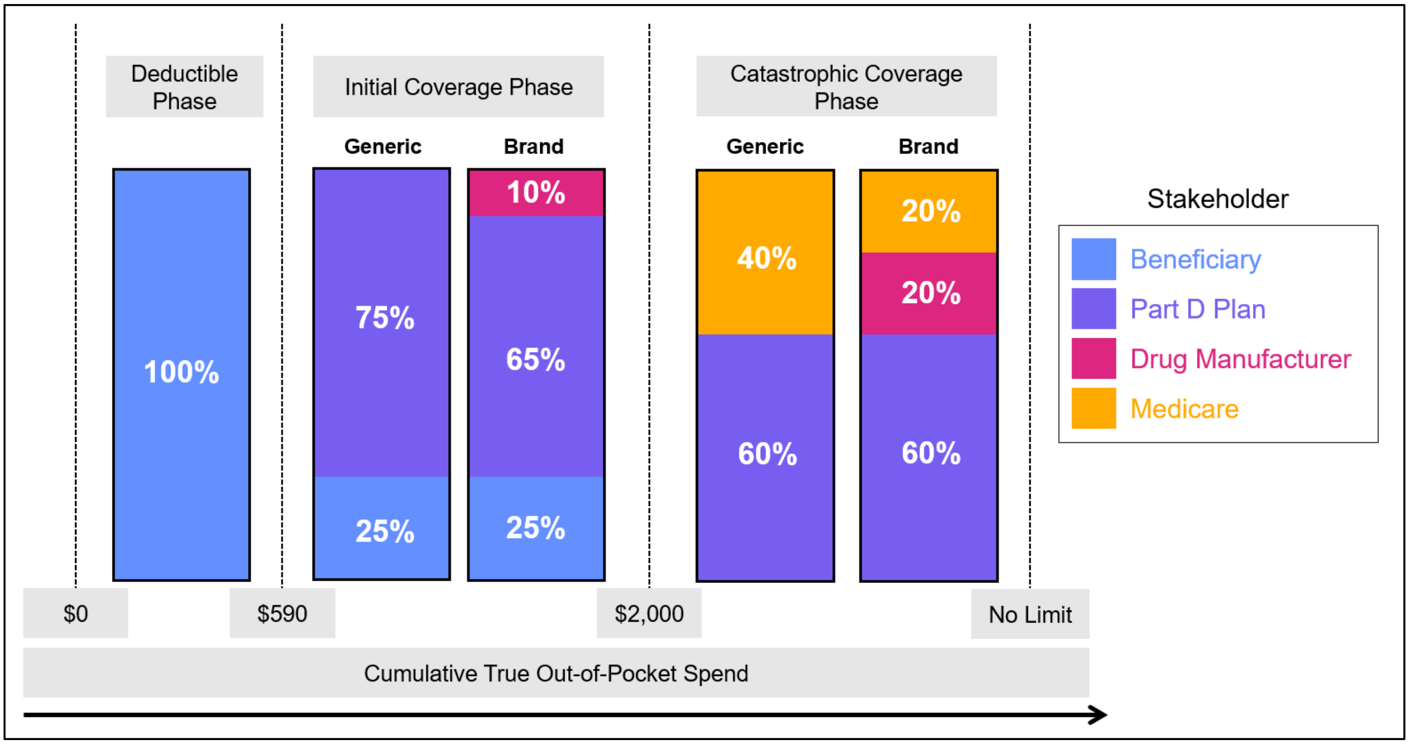

Part D beneficiaries begin the 2025 calendar year with $0 in cumulative True Out-of-Pocket (TrOOP) spend. As beneficiaries fill Part D prescription medications throughout the year, TrOOP spend will begin to accumulate. TrOOP spend for each prescription fill is defined as a combination of 1) beneficiary payments (e.g., co-payments, co-insurance), 2) supplemental benefit payments for those with enhanced alternative Part D plans, and 3) “other” payments from third-party payers such as qualified pharmacy assistance programs or charities. Monthly plan premium payments are not considered in TrOOP calculations.

In the deductible phase ($0 - $590 cumulative TrOOP spend), beneficiaries are responsible for 100% of each Part D medication fill’s cost. In the initial phase ($591 - $2,000 cumulative TrOOP spend), beneficiaries are responsible for 25% of each fill’s cost. The remainder is covered by the Part D plan and, with brand name medications, the drug manufacturer.

For each fill in the catastrophic coverage phase ($2,000 in cumulative TrOOP spend), beneficiaries no longer incur costs. The total cost for each generic fill in this period is split between the Part D plan (60%) and Medicare (40%) as reinsurance. For brand name medications, the Part D plan contributes 60% of total fill cost, followed by 20% from Medicare and 20% from the drug manufacturer.

Cumulative TrOOP spend and Part D phase is reset to $0 (deductible) January 1st of each calendar year.

Method S3. Calculation of Monthly Beneficiary Out-of-Pocket Payment Obligations under the Medicare Prescription Payment Plan (M3P)

Each beneficiary's monthly out-of-pocket payment totals under the Medicare Prescription Payment Plan were calculated as follows:

$$C_t = \begin{cases} \min\left(OOP_t, \dfrac{OOP_0 + M}{12}\right), & if\ t = 1 \\ \min\left(OOP_t, \dfrac{B_{t-1} + OOP_t}{13 - t}\right), & if \displaystyle\sum_{i=1}^{t} C_t \leq M \\ \dfrac{B_{t-1}}{13 - t}, & if \displaystyle\sum_{i=1}^{t} C_t > M \end{cases}$$

Monthly Balance Update:

$$B_t = B_{t-1} + OOP_t - C_t$$

Where:

- t denotes month of participation in the M3P (where t=1 denotes the month beneficiary begins enrollment in M3P).
- C_t denotes the amount charged to each beneficiary in month t under the M3P
- OOP_0 denotes total beneficiary out-of-pocket spending in the year prior to enrollment in the M3P
- M denotes the catastrophic phase coverage limit ($2,000 in 2025)
- OOP_t denotes the beneficiary's out-of-pocket payments at month t (without M3P)
- B_t denotes the amount owed by the beneficiary carried over to the next month under the M3P

Method S4. Analysis of Proportion of Days Covered (PDC) as a Measure of Adherence

We measured beneficiary adherence to any cancer-indicated Part D medication in 2022 using Proportion of Days Covered (PDC). PDC as a measure of adherence was developed by the Pharmacy Quality Alliance (PQA) and has been commonly utilized as a proxy measure of adherence to oral cancer-indicated medications.[1-5] We first measured and defined an “observation period” for each beneficiary, starting from the date of the first claim for a Part D cancer-indicated medication in 2022 until December 31, 2022 or date of death, whichever occurred first. To define PDC with cancer-indicated Part D medications, we extracted all prescription claims for cancer-indicated Part D medications for each beneficiary (Table S1) and arranged them chronologically. Using the date of fill and days’ supply for each prescription claim, we calculated the total number of days a beneficiary should have had access to a cancer-indicated Part D medication (“covered days”), excluding any days past the observation period end date. For each beneficiary, PDC in 2022 was then calculated using the ratio of the number of days “covered” by a cancer-indicated Part D medication in the year (numerator) and the total number of days in the observation period (denominator). We tallied the number of inpatient days per beneficiary, if applicable, and subtracted this value from the observation period prior to calculating PDC. This was done to account for situations where a beneficiary may not have had access to their cancer-indicated Part D medication as part of an inpatient stay. We assumed an acceptable threshold for adherence as PDC of 80% or greater, as has been established in previous research.[1-4]

References:

1. Nau DP. Proportion of days covered (PDC) as the preferred method of measuring medication adherence. *Pharmacy Quality Alliance*. Accessed June 10, 2025. https://www.pqaalliance.org/files/PDCvsMPRfinal.pdf

2. Doshi JA, Jahnke J, Raman S, et al. Treatment utilization patterns of newly initiated oral anticancer agents in a national sample of Medicare beneficiaries. *J Manag Spec Pharm*. 2021;27(10):1457-1468.

3. Vyas A, Descoteaux A, Kogut S, et al. Predictors of adherence to oral anticancer medications: An analysis of 2010-2018 US nationwide claims. *J Manag Care Spec Pharm*. 2022;28(8):831-844.

4.Kaye DR, Wilson LE, Greiner MA, et al. Patient, provider, and hospital factors associated with oral anti-neoplastic agent initiation and adherence in older patients with metastatic renal cell carcinoma. *J Geriatr Oncol*. 2022;13(5):614-623.

5.Zheng D, Thomas J. Survival benefits associated with being adherent and having longer persistence to adjuvant hormone therapy across up to five years among US Medicare population with breast cancer. *Breast Cancer Res Treat*. 2023;201(1):89-104.

Table S3. Demographic Characteristics, Raw

	Total (N=8,424)		Deductible Phase[1] (N=1,997)		Initial Phase (N=2,491)		Catastrophic Phase (N=3,936)	
	N	%	N	%	N	%	N	%
Age (years)								
65-74	3868	45.9	1108	55.5	1119	44.9	1641	41.7
75-84	3430	40.7	693	34.7	1032	41.4	1705	43.3
85+	1126	13.4	196	9.8	340	13.6	590	15.0
Sex								
Female	5490	65.2	1614	80.8	1837	73.7	2039	51.8
Male	2934	34.8	383	19.2	654	26.3	1897	48.2
Race / Ethnicity								
NH, White	7630	90.6	1820	91.1	2267	91.0	3543	80.0
NH, Black	247	2.9	56	2.8	61	2.4	130	3.3
Hispanic	151	1.8	38	1.9	52	2.1	61	1.5
Other	396	4.7	83	4.2	111	4.5	202	5.1
Region								
South	3311	39.3	766	38.4	1054	42.3	1491	37.9
Midwest	1879	22.3	459	23.0	530	21.3	890	22.6
West	1644	19.5	426	21.3	474	19.0	744	18.9
Northeast	1590	18.9	346	17.3	433	17.4	811	20.6
Area-Level Socioeconomic Status (Quartile)								
First (Highest)	2073	24.6	487	24.4	613	24.6	973	24.7
Second	2093	24.8	520	26.0	620	24.9	953	24.2
Third	2053	24.4	494	24.7	598	24.0	961	24.4
Fourth (Lowest)	2205	26.2	496	24.8	660	26.5	1049	26.7
Cancer Type								
Breast	4013	47.6	1385	69.4	1458	58.5	1170	29.7
Dermatologic	1479	17.6	329	16.5	540	21.7	610	15.5
Gastrointestinal	158	1.9	28	1.4	33	1.3	97	2.5
Hematologic	949	11.3	43	2.2	119	4.8	787	20.0
Lung	209	2.5	29	1.5	38	1.5	142	3.6
Miscellaneous	335	4.0	37	1.9	72	2.9	226	5.7
Prostate	1121	13.3	105	5.3	183	7.3	833	21.2
Reproductive	160	1.9	41	2.1	48	1.9	71	1.8
Plan Type								
Enhanced Alternative	6901	81.9	1605	80.4	2079	83.5	3217	81.7
Died	500	5.9	77	3.9	91	3.7	332	8.4
Non-Adherent[2]	3621	43.0	816	40.9	1072	43	1733	44.0

[1]Groups were defined according to total estimated true out-of-pocket (TrOOP) spend in 2025 with the Inflation Reduction Act's Part D cap in place. Deductible: $0-590; Initial Phase: $591-$1,999; Catastrophic: $2,000.

[2]Defined as Percent of Days Covered (PDC) <80% to cancer-indicated Part D medications during calendar year 2022.

Abbreviations: NH: Non-Hispanic; SVI: Social Vulnerability Index

Table S4. Beneficiary Out-of-Pocket Obligations and Adherence by Cancer Type

Cancer Type	Average Annual OOP Obligation (SD)*	% Total Annual OOP Obligation Paid in January (2025)^	% Reaching Catastrophic Coverage (2025)	% Non-Adherent (2022)	% Non-Adherent Reaching Catastrophic Coverage (2025)
Breast	657.6 (578.3)	17.0	29.2	27.8	30.9
Dermatologic	767.7 (560.1)	20.6	41.2	78.6	40.2
Gastrointestinal	1053.5 (687.0)	29.9	61.4	62.0	61.2
Hematologic	1551.7 (616.6)	55.4	82.9	38.7	79.8
Lung	1276.7 (720.9)	41.1	67.9	43.1	62.2
Miscellaneous	1196.1 (689.5)	38.0	67.5	54.6	64.5
Prostate	1381.6 (673.9)	39.7	74.3	47.4	67.1
Reproductive	867.7 (695.5)	26.9	44.4	45.0	52.8

Abbreviations: OOP: Out-of-pocket; SD: Standard Deviation.

*Calculated using mean.

^Percent of total annual out-of-pocket obligations estimated for January are based on a non-Medicare Prescription Payment Plan Scenario.

“Catastrophic Phase” denotes beneficiaries whose total True Out-of-Pocket (TrOOP) costs for 2025 reached the $2,000 cap.

Table S5. Median and Interquartile Range of Monthly Beneficiary Charges, Medicare Prescription Payment Plan (M3P) vs No Medicare Prescription Payment Plan (No M3P), Deductible Phase, 2025

	No M3P			M3P		
Month	Median	IQR	Max	Median	IQR	Max
January	7.1	24.2	572.5	7.1	24.2	166.7
February	4.6	18.2	269.8	0.4	1.8	36.9
March	6.9	23.9	376.0	2.0	4.1	39.2
April	7.9	23.0	361.1	3.7	5.8	43.1
May	5.6	18.8	348.3	5.5	8.1	50.9
June	6.7	20.5	333.4	7.5	11.1	51.4
July	6.4	20.4	339.0	10.0	13.2	70.8
August	5.9	20.7	266.9	12.9	16.5	70.8
September	5.8	19.2	352.1	16.6	19.7	93.6
October	5.8	20.3	308.4	21.7	25.2	120.3
November	4.9	20.7	298.2	27.7	32.6	181.6
December	5.8	20.7	279.4	40.5	48.4	312.3

Abbreviations: M3P: Medicare Prescription Payment Plan; IQR: Interquartile Range

“Deductible group” denotes beneficiaries whose total True Out-of-Pocket (TrOOP) costs for 2025 under the Inflation Reduction Act’s $2,000 TrOOP cap ranged from $0 - $590.

Table S6. Median and Interquartile Range of Monthly Beneficiary Charges, Medicare Prescription Payment Plan (M3P) vs No Medical Prescription Payment Plan (No M3P), Initial Phase, 2025

	No M3P			M3P		
Month	Median	IQR	Max	Median	IQR	Max
January	32.9	93.8	1430.4	32.9	93.8	166.7
February	25.0	75.2	1618.1	3.0	9.0	147.1
March	28.3	81.6	1223.9	9.1	16.0	147.9
April	27.6	75.2	1172.9	15.5	21.8	147.9
May	26.5	75.1	1196.3	22.6	29.3	151.6
June	27.6	73.7	1109.8	31.2	35.8	203.1
July	26.5	66.3	1673.5	39.6	42.4	297.4
August	27.6	71.4	1058.5	50.6	51.0	300.8
September	26.3	69.7	961.5	63.1	59.7	300.8
October	27.6	71.0	711.1	79.9	70.8	300.8
November	25.3	69.4	1044.9	102.7	94.7	598.9
December	27.6	67.1	1770.7	144.5	136.4	1775.1

Abbreviations: M3P: Medicare Prescription Payment Plan; IQR: Interquartile Range

"Initial Phase" denotes beneficiaries whose total True Out-of-Pocket (TrOOP) costs for 2025 under the Inflation Reduction Act's $2,000 TrOOP cap ranged from $591 - $1,999.

Table S7. Median and Interquartile Range of Monthly Beneficiary Charges, Medicare Prescription Payment Plan (M3P) vs No Medical Prescription Payment Plan (No M3P), Catastrophic Phase, 2025

	No M3P			M3P		
Month	Median	IQR	Max	Median	IQR	Max
January	301.8	1798.4	2000.0	166.7	118.4	166.7
February	23.2	183.5	2000.0	52.8	156.2	181.8
March	2.2	124.3	2000.0	72.8	142.7	200.0
April	0.0	67.4	2000.0	90.0	131.7	222.2
May	0.0	37.2	2000.0	107.7	120.6	250.0
June	0.0	8.0	2000.0	121.2	111.1	285.7
July	0.0	0.0	2000.0	135.9	103.7	333.3
August	0.0	0.0	1979.4	148.7	97.5	397.8
September	0.0	0.0	1962.8	156.8	92.9	493.6
October	0.0	0.0	2000.0	161.6	87.9	666.7
November	0.0	0.0	1980.9	162.2	87.6	992.0
December	0.0	0.0	2000.0	163.3	87.8	2000.0

Abbreviations: M3P: Medicare Prescription Payment Plan; IQR: Interquartile Range

“Catastrophic Phase” denotes beneficiaries whose total True Out-of-Pocket (TrOOP) costs for 2025 reached the $2,000 cap.

Table S8. Demographic Characteristics Among Beneficiaries with 2025 Part D Claims Containing Out-of-Pocket Obligations of $600 or Greater

	No Beneficiary Claim OOP Obligation ≥$600[1]		Beneficiary Claim OOP Obligation ≥$600	
	N	%	N	%
Expected 2025 Part D Benefit Phase[2]				
Deductible	1,997	33.0	0	0.0
Initial	2,370	40.0	121	4.8
Catastrophic	1,558	26.3	2,378	95.2
Cancer Type				
Breast	3,454	58.3	559	22.4
Dermatologic	1,246	21.0	233	9.3
Gastrointestinal	93	1.6	65	2.6
Hematologic	274	46.3	675	27.0
Lung	97	1.6	112	4.5
Miscellaneous	181	3.1	154	6.2
Prostate	464	7.8	657	26.3
Reproductive	116	2.0	44	1.8
Average Annual Beneficiary OOP Obligation (SD)	570.7 (466.1)		1,755.4 (361.8)	
Adherence Status (2022)[3]				
Yes	3,341	56.4	1,462	52.5
No	2,584	43.6	1,037	41.5

Raw sample sizes are reported.

[1]Beneficiaries were classified as having a claim OOP obligation of $600 or greater if they had at least one claim with an expected OOP obligation of $600 or more assuming a $2,000 true out-of-pocket spend limit under the Inflation Reduction Act.

[2]Groups were defined according to total estimated true out-of-pocket spend in 2025 with the Inflation Reduction Act's Part D cap in place. Deductible: $0-590; Initial Phase: $591-$1,999; Catastrophic: $2,000.

[3]Defined as Percent of Days Covered (PDC) <80% to cancer-indicated Part D medications during calendar year 2022.

Abbreviations: OOP: Out-of-Pocket; SD: Standard Deviation
